# Complex Above- and Below-Ground Growth Responses of Two Urban Tree Species Following Root, Stem, and Foliage Damage—An Experimental Approach

**DOI:** 10.3389/fpls.2019.01100

**Published:** 2019-09-18

**Authors:** Valentina Vitali, Jorge A. Ramirez, Guillaume Perrette, Sylvain Delagrange, Alain Paquette, Christian Messier

**Affiliations:** ^1^Faculté des sciences, Département des sciences biologiques, Centre d’Étude de la Forêt (CEF), Université du Québec à Montréal, Montréal, Canada; ^2^Facultad de Ciencias Agrarias, Universidad del Cauca, Popayán, Colombia; ^3^Institut des Sciences de la Foret Tempérée, Université du Québec en Outaouais, Ripon, Canada

**Keywords:** *Celtis occidentalis*, damage and stress, *Fraxinus pennsylvanica*, tree growth, urban environment

## Abstract

Urban trees are subjected to numerous biotic and mechanical damages, which can affect their growth rates and health. However, for most species, a systematic analysis of tree above- and below-ground growth reactions to a variety of damages is still lacking. Under a fully factorial experimental setup, using two common urban trees (*Celtis occidentalis, Fraxinus pennsylvanica*), we tested the effects of various degrees of frequently occurring damage as defoliation, root reduction, and stem injuries for a total of 18 treatments. We hypothesized that (i) an increasing amount of damage would proportionally negatively affect both root and stem growth; (ii) there would be a lag or lasting effect on growth; and (iii) both species would react similarly to the treatments. Contrary to our expectation, increasing levels of single or combined damage did not have an incremental effect on either stem or root growth. Although *Celtis* was significantly less vigorous than *Fraxinus*, it did not react strongly to damage treatments compared to the control. Interestingly, *Celtis* that experienced stem damage alone or in combination with other damages showed higher growth rates than the control. For *Celtis*, root injury was the treatment having the most impact, decreasing both root and stem growth consistently throughout the 5 years following treatments, whereas defoliation decreased growth only in the first 2 years. All damage treatments negatively affected stem and root growth of *Fraxinus* trees. Stem growth was affected the most by defoliation in the first year following the treatment, while root injury became the driving factor in subsequent years. For both species, stem injury showed the least influence on growth rates. The control and low-level damage treatments often affected growth rates in a similar way, suggesting that low-intensity stress triggers compensatory reactions stimulating photosynthetic rates and nutrient utilization. The slower-growing tree species, *Celtis*, showed a less negative reaction to all damage treatments compared to *Fraxinus*. This study illustrates that various types of above- and below-ground injuries do not have a simple additive effect on tree growth and that trees are capable of compensating for the loss of foliage, roots, or phloem to meet their metabolic demand.

## Introduction

Trees growing in urban landscapes are an invaluable asset as they provide numerous environmental, social, cultural, and economic benefits (Konijnendijk et al., 2005; Tanner et al., 2014). Yet, urban environments are often characterized by low water availability, higher temperatures induced by the urban heat island phenomenon, and limited space (Sieghardt et al., 2005; Moser-Reischl et al., 2018). All too frequently, urban trees are also damaged by bystanders or during construction or maintenance work on urban infrastructure. Consequently, these trees have to withstand a wide variety of damages and accidental injuries, such as root trenching for road work or sidewalk placement or reparation; trunk scarification damage caused by construction and infrastructure maintenance; and crown reduction or defoliation through pruning, drought stress or insect infestation (Clair-Maczulajtys et al., 1999; Millet and Bouchard, 2003; Smiley, 2008; Jacquet et al., 2012). Such levels of damage and stress, alone or in combination, can dramatically affect tree growth and vitality and lead to mortality, thereby increasing costs for removal and replacement.

Such deviation from the typical growth environment of trees affects several physiological mechanisms, which can lead to drastic changes in growth rates in different parts of the tree (Boege, 2005; Jacquet et al., 2014; Freschet et al., 2018). Thus, tree growth is maximal when both above- and below-ground environmental conditions and resources are optimal and declines when these values are suboptimal (Niinemets and Valladares, 2008; Niinemets, 2010b). Overall, tree species that have the capacity to tolerate the most extreme environmental conditions have low plasticity and low growth rates (Niinemets, 2010b), possibly due to the associated carbon cost of maintaining a positive carbon balance during acclimation (Ramirez, 2017)

Trees growing in urbanized conditions face several stressors that can act alone or in combination (Jutras et al., 2010; Calfapietra et al., 2015) and can generate a unique set of responses due to a mitigation effect (Mittler, 2006; Niinemets, 2010a). Their growth response may be more severe due to negative interactions between stressors or less severe due to mitigation effects (Mittler, 2006; Niinemets, 2010a). Although there is a strong interest in ensuring vigorous and healthy trees in urban settings, our understanding of urban tree reactions to injury, and in particular to a combination of injuries, is still poor (Niinemets, 2010a; Ferrini et al., 2014). In this study, we focus on single and combined effects of three types of injuries, which typically affect urban trees: defoliation, root reduction, and stem damage.

Defoliation can cause a decrease in tree photosynthetic capacity, reducing available carbon for growth, and build-up of reserves (Eyles et al., 2009; Pinkard et al., 2011). This damage can also activate short- and long-term physiological mechanisms including the reduction of biomass allocation to coarse roots (Eyles et al., 2009) and the mobilization of carbohydrate reserves from branches, stems, and roots to increase the metabolism of remaining leaves to compensate for the decreased supply of carbohydrates (Quentin et al., 2010). Root reduction affects the hydraulic system and water uptake, reducing the amount of water available for transpiration and further affecting photosynthesis, while actively removing storage organs (Vysotskaya et al., 2004). Stem damage impacts both water and sugar translocation between above- and below-ground organs affecting reserves build-up and photosynthetic rates (Fajstavr et al., 2017) and, in extreme cases, can induce wilting when the damage reaches deep into the xylem and water transportation is drastically reduced (Moore, 2013), or even tree death by starvation when translocation of sugars is strongly decreased (Adams et al., 2013; Oberhuber et al., 2017). After a stress episode, different tree species will show different capacities to recover from the damage and maintain or increase growth rates. This capability is based on life history traits of the species and its resource allocation to growth and defense (Grime and Hunt, 1975). It is expected that functionally different species will exhibit variation in carbon allocation and therefore in recovery after damage (Poorter and Kitajima, 2007).

All injuries affect physiological mechanisms, which can be evaluated through changes in growth rates in different parts of the tree. It is expected that these injuries lead to shifts in the carbon allocation priorities to maintain their metabolic activities and to start compensatory growth. In this study, we will use tree-ring data to assess the effect on growth and resilience to multiple kinds of mechanical damage, single and combined, in two commonly used urban species in Eastern North America: North American hackberry (*Celtis occidentalis L.*) and green ash (*Fraxinus pennsylvanica* Marsh.). Furthermore, these species show distinctly different resource utilization strategies; where the North American hackberry shows a lower growth speed and a conservative use of resources (i.e., photosynthetic products), ash is growing faster and uses resources more intensively (Ramirez, 2017). We will compare the growth response in stem and roots of both species to determine whether biomass allocation above- and below ground was altered by various types and levels of damage treatments, and if there was a lag or lasting effect on growth rates in the years after the damage, treatments were applied to address the following hypotheses:

1. An increase in damage intensity results in a simple additive negative growth response:
  - Main stem and large roots show greater declines in growth when treated with multiple types and high damage intensities.
  - The combination of both root reduction and defoliation causes lower decline of overall tree growth at independently of the treatment intensity.
2. Species strategies to compensate for damages change with species-specific physiology. A resource-conservative species (*Celtis*) will be less affected by mechanical damages than a resource-intensive species (*Fraxinus*).
3. Above- and below-ground growths of the two species return to pretreatment levels in the years after the treatment.

## Methods

### Study Site

This study was conducted in the municipal nursery of the City of Montreal, province of Quebec, Canada. The site lies at 45°49′59″ N, 73°24′44″ W, at about 35 m of elevation. The mean annual precipitation is 978 mm (215 mm snow and 763 mm rain), and the mean annual temperature is 6.2°C (mean growing season temperature is 14.4°C). The mean daily maximum and minimum temperatures are 10.5°C and 0°C. Annually, there is an average of 1,958 h of sunshine, 2.8 degree-days below 10°C, and 1,099 degree-days above 10°C. Temperatures below 0°C occur between October and April (period 1971–2000; Environment Canada, climate.weather.gc.ca) and the winter season commonly extends from mid-November to the end of March (Boyer et al., 1985). Soil structure is mainly composed of a loamy clay.

### Study Species

**Figure 1:**
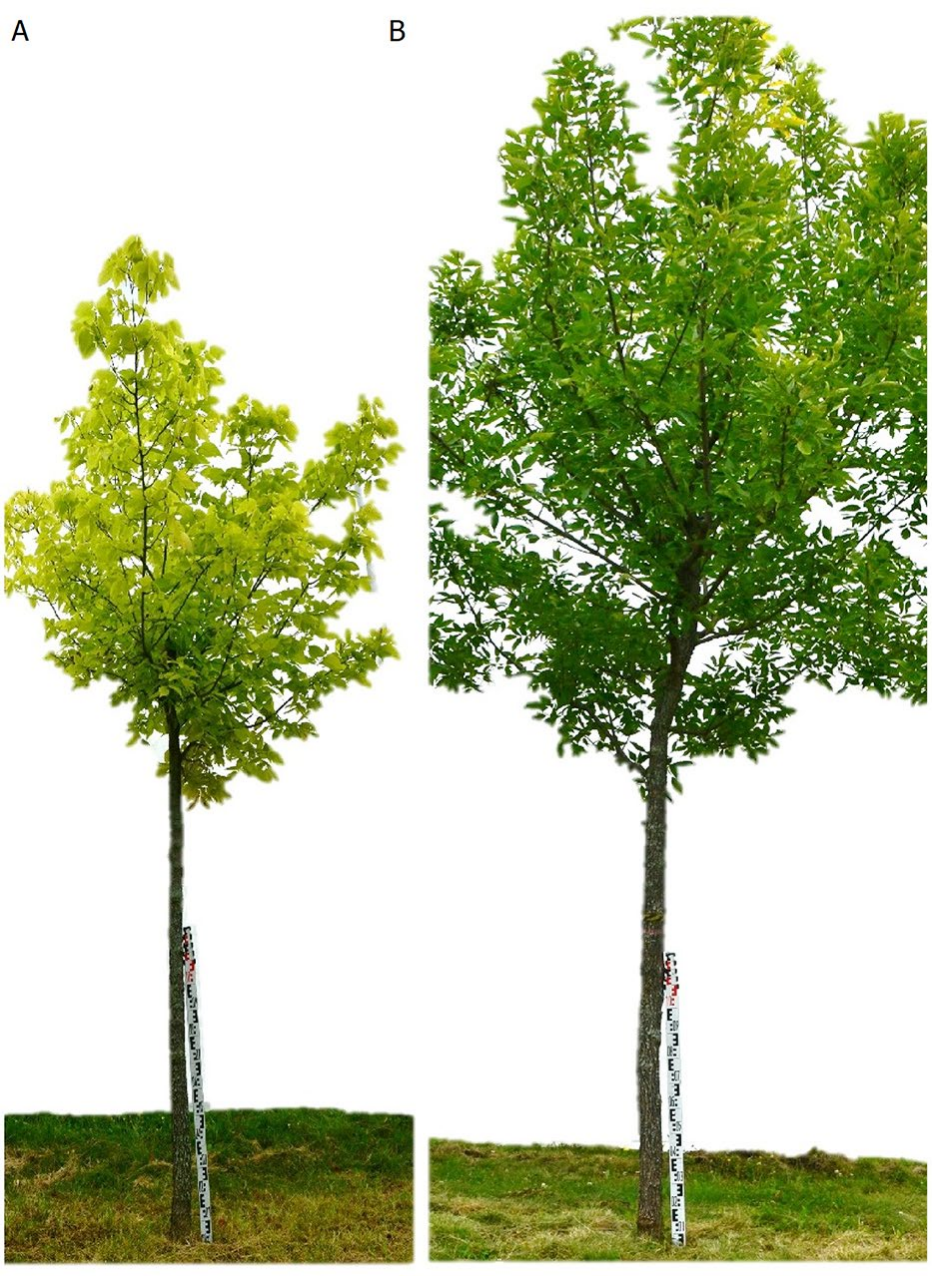
Examples of control trees of **(A)** *Celtis* and **(B)** *Fraxinus* taken at the end of the experiment period in 2017. Ruler for scale is 1m in length.

We studied two tree species commonly planted in the city of Montreal: *C. occidentalis* L. (common hackberry) and *F. pennsylvanica* Marsh. (Red ash), hereafter referred to as *Celtis* and *Fraxinus*, respectively. Both species show a large variability across their geographical range, but they are generally characterized as medium-sized (*Celtis* reaching 15 m and *Fraxinus* 25 m in height), with a medium lifespan (100 years for *Fraxinus* and 150 years for *Celtis*), and are in generally considered fast growing, although they show distinctively different growth rates (Farrar, 1995). *Celtis* is a more resource conservative species since it has a higher foliar carbon/nitrogen ratio, lower photosynthetic capacity, and higher wood density than *Fraxinus* (Ramirez, 2017). Moreover, *Celtis* is capable of growing in very inhospitable conditions (Gucker, 2011), while *Fraxinus*, although showing a broad climate adaptability, prefers growing in mesic to humid sites (Gucker, 2005). The exemplars for this experiment were donated by the City of Montréal. At the seedling stage, there were no apparent differences in vigor between the species. However, after e-planting, *Celtis* showed signs of growth decline independent from the application of treatments. Trees at the beginning of the study (2012) were between 1.6 and 4.5 m tall for *Celtis*, and 2.6 to 6.1 m tall for *Fraxinus*. Therefore, the results presented hereafter compare both two different species and two states of vigor (**Figure 1**). A total of 202 trees were planted in 2009 and 2010, 116 *Celtis* from field-grown seedlings propagated in 2004, and 86 *Fraxinus* seedlings propagated in 2003.

### Experimental Design: Damage Treatments

**Figure 2:**
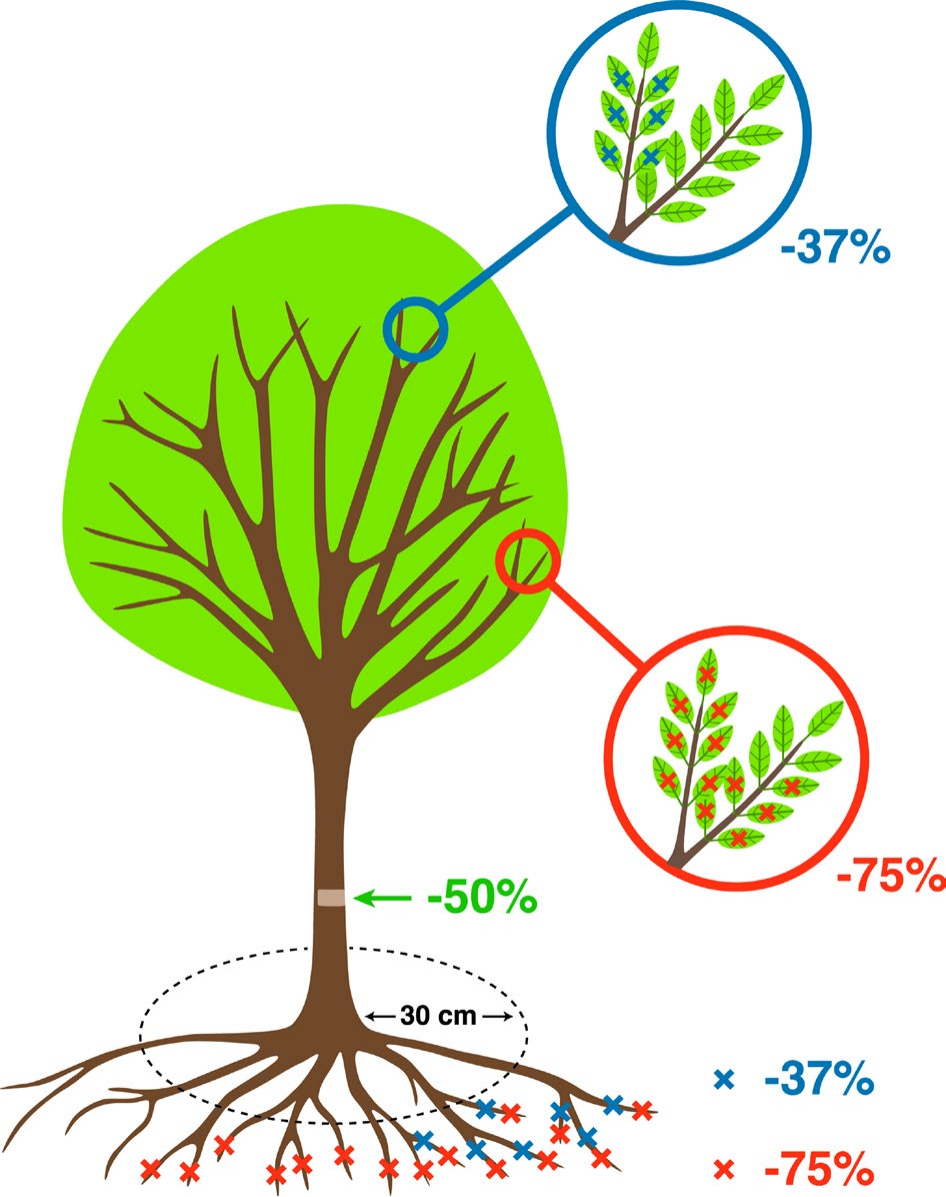
Schematic representation of the treatments described in **Table 1** (more details about stress treatments in Ramirez (2017).

A fully factorial experiment was set up; where in addition to the control with no damage treatment, all other trees were randomly assigned to the following damage treatments: two intensities of defoliation (37%, 75%), two intensities of root reduction (37%, 75%), and one intensity of stem damage (50%) (**Table 1**, **Figure 2**). All possible combinations among these three treatments were reproduced, resulting in 18 types and levels of damage treatment. The number of replicates per treatment was 6 in *Celtis* and 4 in *Fraxinus* due to the availability of trees, and this varied slightly between treatments to ensure sufficient trees in case of mortality. Treatments were applied in July, which corresponded to the month of maximum leaf area in both 2012 and 2013. At the beginning of the dormant season in autumn 2015, a reduction pruning of the main stem was performed on all trees with the removal of the lower half of the living crown. This treatment was applied to all trees, but its effect will not be investigated in this instance as analyses specific to crown recovery and architecture have been carried out in a separate study.

**Table 1:** Description of the damage treatments performed in 2012 and 2013 [more details about stress treatments in Ramirez (2017)]. For *Fraxinus*, we used six trees per treatment on average, with the exception of nine for the maximum damage treatment group, while *Celtis* on average had four trees per treatment and eight for the maximum damage treatment group.

Treatment		Method	Intensity
**DEFOLIATION**	(DF)	Manual removal of leaves at the base of the petiole for all branches	High = 75% Low = 37% Control = no treatment
**ROOT REDUCTION**	(RR)	Tree spade machine cutting at 30-cm radius from stem base	High = 75% Low = 37% Control = no treatment
**STEM DAMAGE**	(SD)	40-mm-wide strip removal, 30 cm above root base. Only cambium and phloem connection were removed	Damage 50% Control = no treatment

### Sample Collection and Dendrochronological Measurements

**Figure 3:**
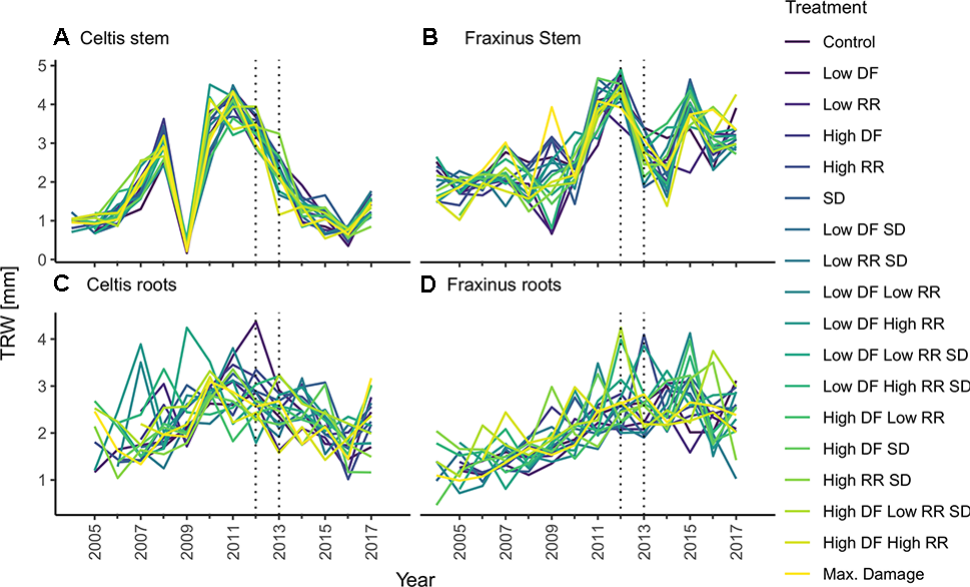
Tree-ring chronologies of **(A)** *Celtis* stem growth, **(C)** *Celtis* average root growth, and **(B)** *Fraxinus* stem growth, and **(D)** *Fraxinus* average root growth for each combination of damage treatments. Low growth in 2009 for *Celtis* and 2009 and 2010 for *Fraxinus* is related to replanting operations. Damage treatments were applied in 2012–2013 as indicated by the two vertical dotted lines. Damage treatment levels are described as follows: DF, defoliation; RR, root reduction and SD, stem damage indicated as low = 37% damage and high = 75% damage.

For each tree, the stem discs were collected at 1.3-m height, and three large roots were cut at 5 cm from the stem base in winter 2017. These cross sections were left to air dry and subsequently sanded down to 400 grit sandpaper (Schweingruber, 1988). Tree ring widths were measured with WINDENDRO^TM^ (Regents Instruments, Quebec). Data quality of single tree chronologies was controlled through visual and statistical cross dating (i.e., gleichläufigkeit). Cross-dating analyses were performed with the dplr package (Bunn, 2010), and mean tree chronologies were created from the average of the three radiuses per cross section from stem and roots (**Figure 3**). All computations were performed using R version 3.4.3 (R Core Team, 2017). Considering the short time span analyzed and the even age status of the trees, we decided to perform our calculations on both raw tree ring widths and on converted basal area increment (BAI) data, obtained with the function bai.out function in the dplr package (Bunn, 2010). This decision was to ensure the preservation of small year-to-year variability that could be potentially concealed through standardization of the chronology (Fritts, 1976).

It is to be noted that *Celtis* showed a replanting shock reaction in 2009, while *Fraxinus* showed a smaller growth decline due to replanting, which occurred in 2009 and 2010. Overall, it should be observed that controls for *Celtis* showed a declining growth rate, while *Fraxinus* showed a vigorous growth trend. Therefore, the effect of the treatments should be related to the actual growth trends of the two species, and the results presented are relative to these growth trends.

### Data Analyses

#### Mixed Models: Overall Treatment Effects

To evaluate the effects of each treatment, alone and in combination with other damage treatments, on the growth response of stems and roots for each species, we created a mixed model that considered growth data from the years 2012–2015.

(Eq.1) $$G_{i\text{j}}=\beta_{0\text{i}}+S_{0\text{ij}}+\beta_{1\text{i}}X1_{\text{ij}}+e_{\text{ij}}$$

where *G*_ij_ is the response variable (tree ring width of stem and root samples for each tree in each year), β_0_ is the intercept, β_1_ is the parameter estimate, *S*_0ij_ is the random effect (for consecutive years measurement and tree identity), X1 is the fixed effect representing the treatment level, which ranges from the control (damage = 0) to the maximum damage (where damage SD = 50%, DF = 75%, RR = 75%), and *e*_i_ is the error. All computations were performed with the lme4 package (Bates et al., 2015).

#### GBM Model: Year-by-Year Treatment Effects

Boosted regression tree (GBM) models were used to evaluate the effect and relative importance of every single damage treatment on the annual growth of *Celtis* and *Fraxinus*. Boosted regression trees (GBM models) have important advantages for tree-based statistical methods. They can handle different types of predictor variables, cope with small sample sizes, and automatically handle interaction effects between predictors (Cutler et al., 2007; Elith et al., 2008; Olden et al., 2008).

The learning algorithm for additive GBM is based on the sequential building of “weak” trees, built atop some randomly chosen variables, which are fitted simultaneously, improving the model with every iteration. Each GBM analysis was based on the mean squared loss function; analysis of variance (ANOVA) model with three-way interactions; 1,000 trees; 5-fold cross validation (Friedman, 2002). Next, the best of these models was chosen, based on the residual sum of squares criterion (Friedman, 2001; Natekin and Knoll, 2013). The calculations were performed with the gbm.step (gbm package) function, and the learning rate and bag fraction were kept constant between the models (Ridgeway et al., 2019).

To visualize the results of the GBM model, we created two types of plots: relative variable influence plots and partial dependence plots. The former shows how important each treatment is to growth, but it does not provide any explanation about how the variable affects the response. The relative influences were further standardized to add up to 100% so that each of the treatments could explain a percentage of the growth rates. The partial dependence plots subdivide the contribution of each level of treatment to the estimated growth. The partial dependence plots display the average change in predicted growth as we vary the effect of each treatment while holding all other variables constant. Summing up the estimate for any combination of DF, RR, and SD levels will determine the estimated growth for that treatment.

#### Above- and Below-Ground Biomass and Root Architecture in 2017

The entire root system from all trees was excavated mechanically in autumn 2017 allowing for a complete assessment and measurement of fine and larger structural roots. All roots with diameter greater than 3 mm were measured and inventoried for the calculation of roots total diameter increment. The presence of the finer roots was recorded as percent cover within the inner 20-cm radius of the stump center. To assess differences between the intensities and combinations of damage treatments and the control, ANOVA and *post hoc* Tukey tests were used. A simple linear model was used to evaluate the above- and below-ground diametric growth:

(Eq. 2) $$Y_{i}=\beta_{0}+\beta_{1}Z1_{i}+e_{i}$$

where *Y*_i_ is the sum of all measured root diameters for each tree *i*, β_0_ is the intercept, and β_1_ is the parameter estimates of Z1, which is the diameter at breast height (DBH) value in 2017. All computations were performed using R version 3.4.3 (R Core Team, 2017).

To compensate for the root measurement threshold of 3 mm, fine root abundance was estimated for the 20-cm radius area surrounding the stump. In this zone, the area covered by fine roots was visually estimated, where 5% indicates an almost absence of fine roots, and 90% to 100% a dense reticulum of fine roots.

## Results

### Mixed Models to Evaluate Overall Effects for All Years

**Figure 4:**
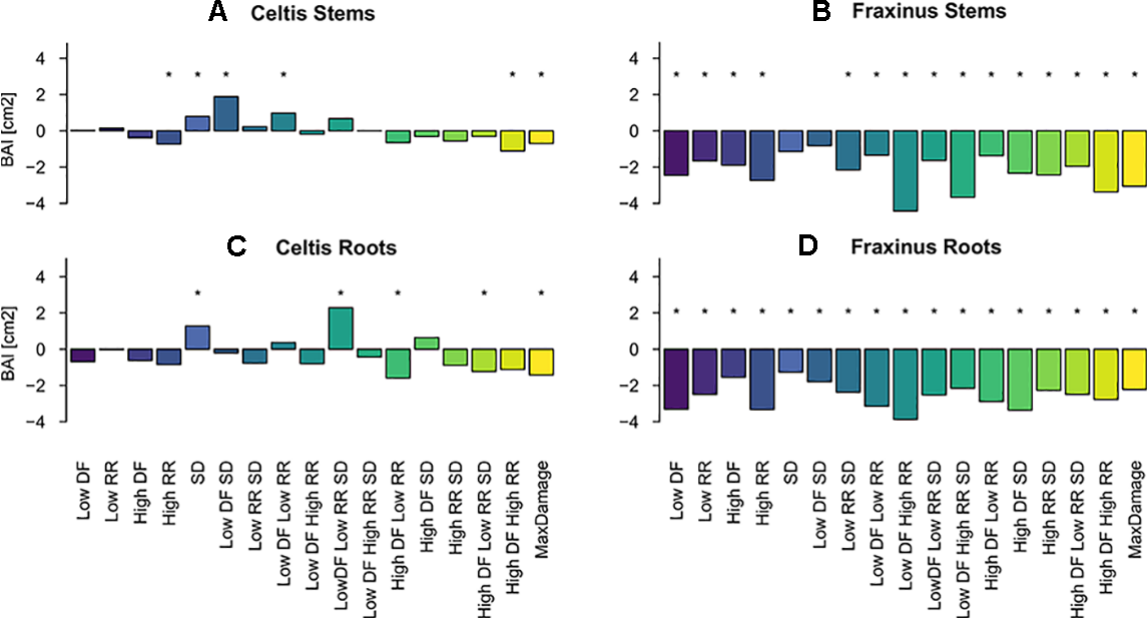
Estimated coefficients of the linear model effect sizes for basal area increment (BAI) growth of *Celtis* (**A** for stem and **C** for roots) and *Fraxinus* (**B** stem and **D** for roots) in the period 2012–2015 (years following last damage treatment), and in relation to the damage treatment level (1–18 treatments are color-coded; DF, defoliation; RR, root reduction; and SD = stem damage, indicated as low = 37% damage and high = 75% damage). *r*² ∼0.1 for all models. For simplicity, asterisks in the figure indicate a significant effect of the variable for *P* < 0.05, discrimination between significance levels can be found in **Table S.1**. The standard deviations for the random effects for the year and the tree id were *Celtis* stems: 0.9 and 1.09, *Fraxinus* stems: 1.53 and 1.8, *Celtis* roots: 0.3 and 1.9, *Fraxinus* roots: 0.76 and 1.75.

Overall, both stem and root growth of *Celtis* were less affected by the various levels and combinations of damage treatments than *Fraxinus*, showing few significant growth differences when compared to the control; in some cases, the stem damage treatment stimulated growth (**Table S.1** and **Figures 4A, C**). On the contrary, stem and root growth of *Fraxinus* were, in general, significantly negatively affected by most damage treatments, both alone and in combination, especially the combinations of defoliation and root damage (at both high and low intensities) where it had the lowest stem growth (**Table S1** and **Figures 4B, D**). *Fraxinus* root growth was consistently reduced by all damage treatments. Only following SD and low DF-SD treatments, *Fraxinus* did show a nonsignificant decrease in growth compared to the control.

### Results GBM Model: Year-by-Year Treatment Effect

**Figure 5:**
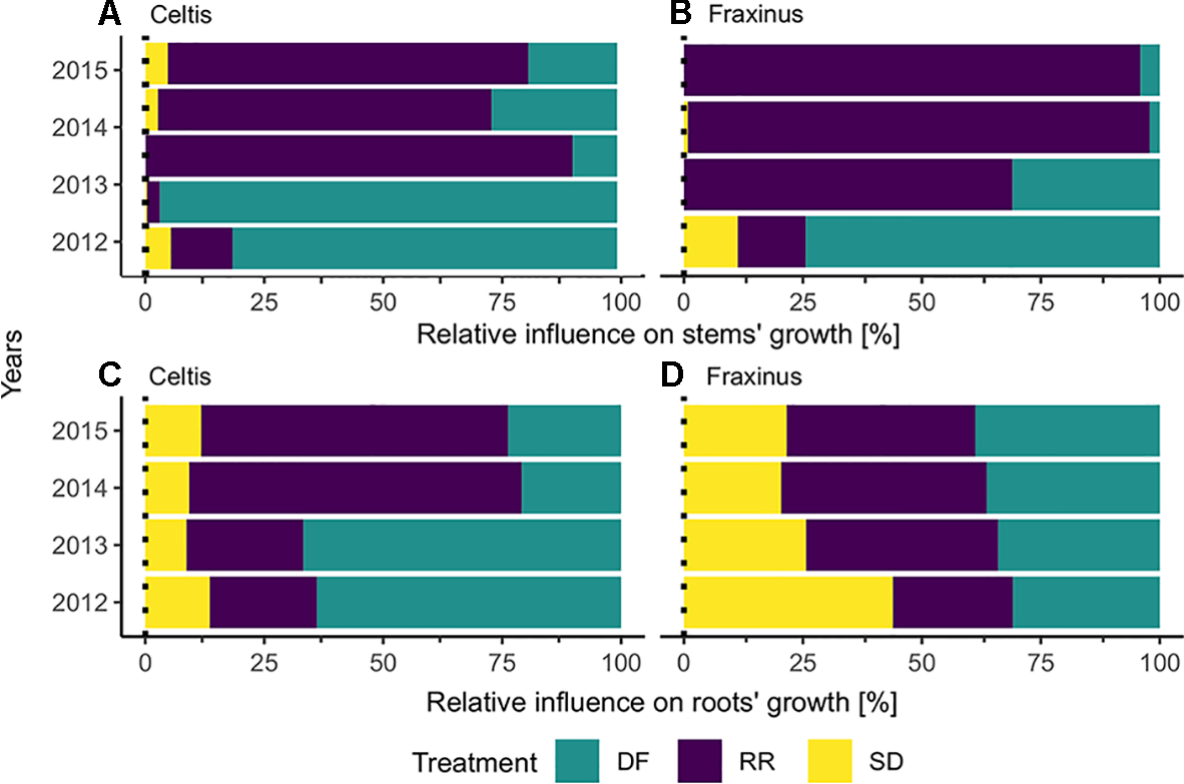
Results from the boosted regression trees: relative influence of each treatment on **(A)** *Celtis* and **(B)** *Fraxinus* stems, **(C)** *Celtis*, and **(D)** *Fraxinus* roots, in the years following damage treatment. DF, defoliation; RR, root reduction and SD, stem damage.

The results calculated by the GBM models indicating the effect of the three damage treatments in the years after they were applied are shown in **Figure 5**. Defoliation had the strongest impact on both *Celtis* and *Fraxinus* stem growth in the 2 years after the damage treatments. However, in the subsequent years, root reduction had the strongest impact. Stem damage had only a minor effect on stem growth (**Figures 5A, B**), but it had a stronger impact on root growth for both *Celtis* and *Fraxinus* throughout the years (**Figures 5C, D**). *Celtis* root growth was more strongly impacted by defoliation than by root reduction in the 2 years after the damage treatments and vice versa in the later years, while the impact of defoliation and root reduction on *Fraxinus* root growth was similar in all years.

**Figure 6:**
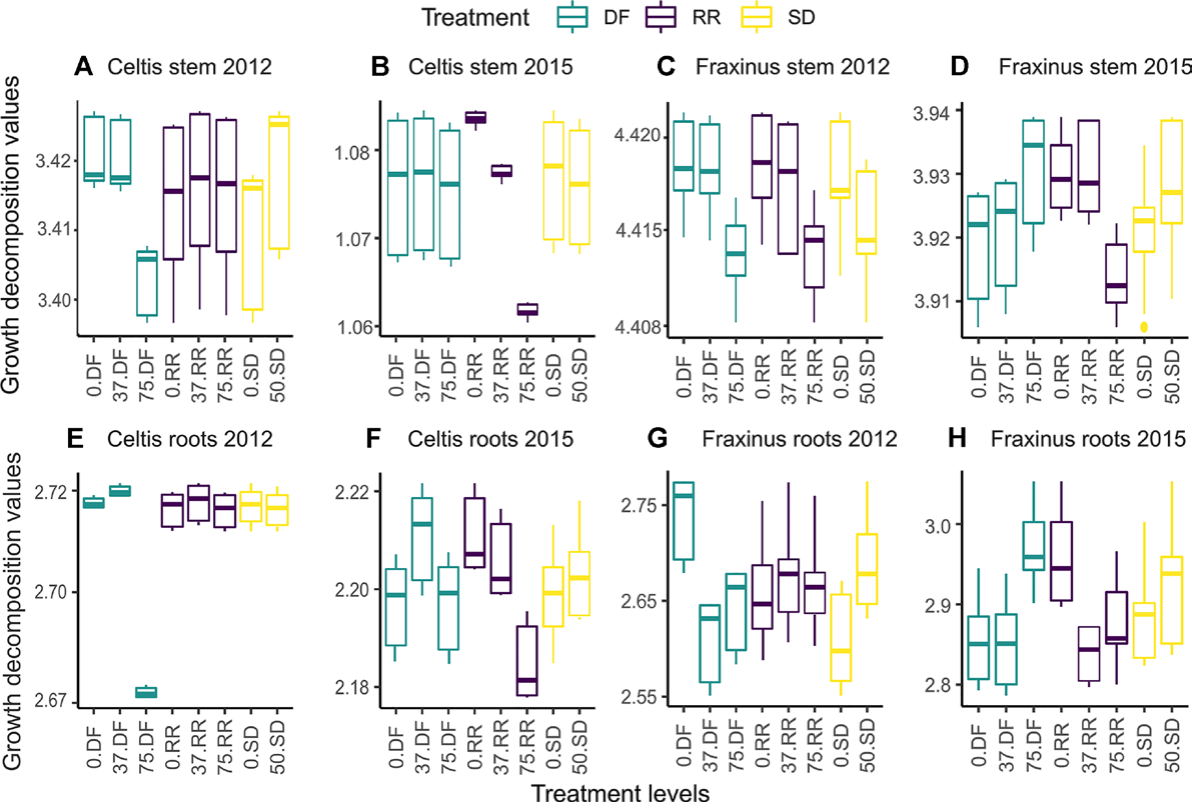
Partial dependence plots showing tree ring growth decomposition between the different levels of damage treatments obtained by the GBM model for the first year (2012) and last year (2015) following damage treatment. DF, defoliation; RR, root reduction; and SD = stem damage, 0 = null treatment, 37 = low intensity treatment, 75 = high intensity treatment. Panels **(A** and **B)** for the stem growth of *Celtis*, **(C** and **D)** for the stem growth of *Fraxinus*, **(E** and **F)** for the root growth of *Celtis*, and **(G** and **H)** for the root growth of *Fraxinus*. Scales are different in each panel to maximize the visibility of the differences between the treatment levels. The vertical lines represent the “whiskers” for the 5th and 95th percentiles of the data distribution.

The marginal effects of each treatment level on the decomposition of stems (**Figure 6D**) and root growth (**Figures 6E–H**) for both species in the first year (2012) and last year (2015) are shown in **Figure 6**. The variable with the highest relative influence is also the one accounting for the greatest differences in growth; when a variable is more influential, the fitted growth shows a positive or negative trend depending on the effect of the damage treatment (i.e., from 0 = control, low = 37% damage, and high = 75% damage, see **Table 1**). The mean annual growth for each treatment can be calculated by summing the GBM fitted results in **Figure 6** for the respective treatment level.

High defoliation in *Celtis* showed the lowest contribution to growth rates in 2012, while low defoliation and no defoliation damage treatments had similar contribution rates (**Figure 6A**). On the contrary, in 2015, root reduction played the biggest part in differentiating growth rates, which was indirectly proportional to its intensity (**Figure 6B**). Similar patterns can be observed for *Celtis* root growth; however, in 2015, the differences between the damage treatment levels are more evident (**Figures 6E, F**). Interestingly, in this case, low defoliation shows higher growth than the null and high damage treatments, while root reduction contribution is still decreasing with increasing intensity of damage treatments (**Figure 6F**).

The decomposition of *Fraxinus* growth shows a more even distribution among the damage treatments. Interestingly, the low intensity DF and RR damage treatments show a more similar effect than their control counterparts for stem growth (**Figures 6C, D, H**). The trend of the DF contribution to growth drastically changes in 2015, showing a higher contribution to growth from high DF than the null and low intensity damage treatments (**Figures 6D, H**), while high intensity RR shows the lowest contribution to the growth of roots (**Figure 6E**). For both species, in most cases, the SD treatment has either an equal or a higher contribution than the no stem damage.

### Stem Size, Larger Root Area, and Root Architecture in 2017

**Figure 7:**
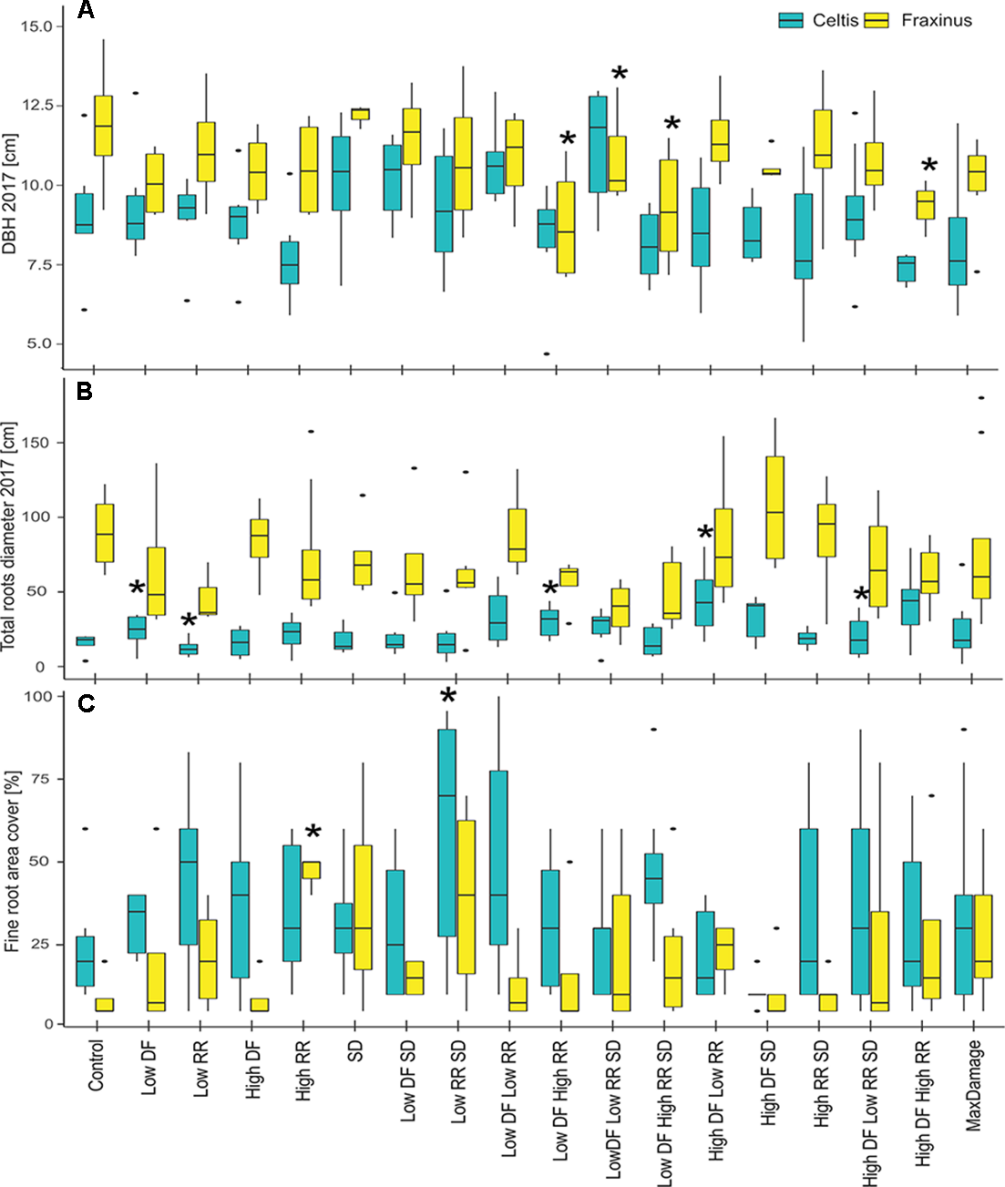
**(A)** DBH measurements in 2017, **(B)** total larger roots area, measured at 20 cm from the stem center, and **(C)** percent area covered by fine roots for the two tree species and all damage treatments, alone and in combination. All damage treatments are compared to the control group for the respective species. Significant difference performance is indicated by an asterisk (ANOVA *P* > 0.05). The difference between the two species growth is also significant in all three cases.

In 2017, the final DBH and the total root area cover showed a nonsignificant difference between the damaged trees and the controls in most cases (**Figures 7A, B**). DBH in *Fraxinus* trees was significantly lower than control in low DF/high RR, low DF/high RR/SD, and high DF/high RR treatments. *Celtis* showed no significant differences in DBH but showed a higher growth of roots in the trees with low DF/low RR, low DF/high RR, high DF/low RR, and high DF/high RR/SD than in the control. There was an increase in production of finer roots with increasing intensity of the damage treatments, particularly for the low RR/SD treatment in *Celtis*, and high RR in *Fraxinus* (**Figure 7C**).

**Figure 8:**
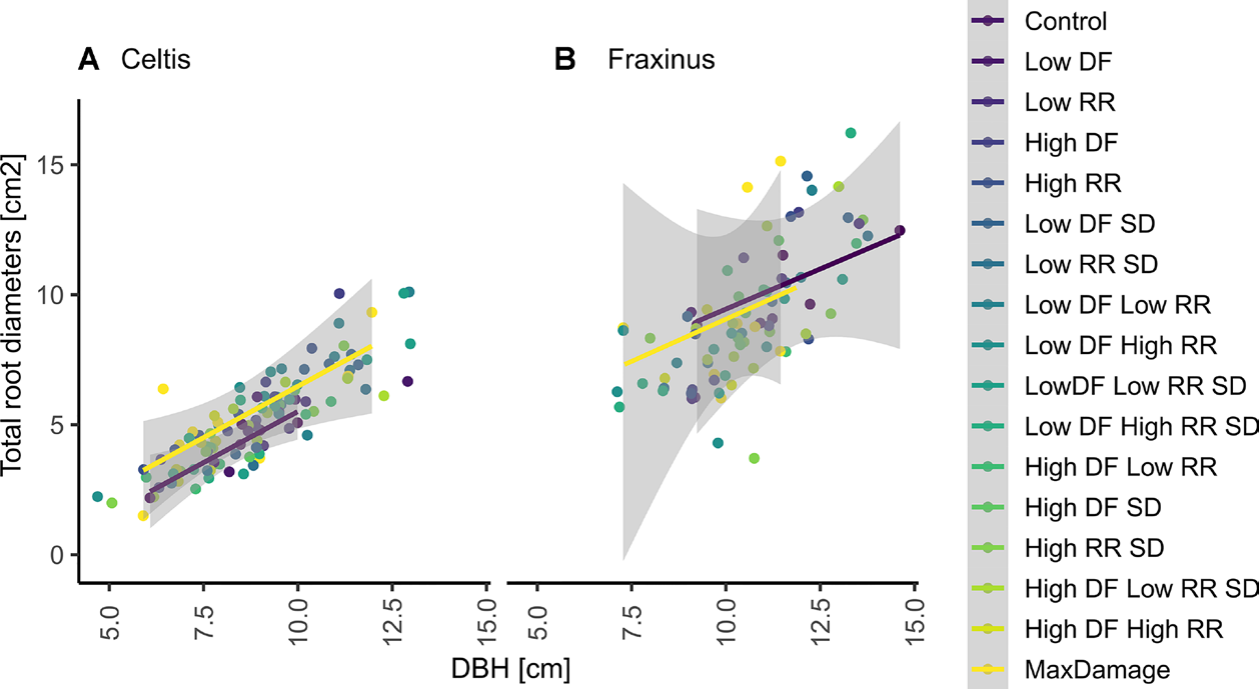
Correlation between DBH and the sum of all larger root diameters at 20 cm from the stump center in 2017 on **(A)** *Celtis* and **(B)** *Fraxinus*. The fitted linear model shows a positive linear correlation with the control group (blue line) and with the maximum combination of damage treatment (yellow line) where *r*^2^ = 0.7 for *Celtis* and *r*^2^ = 0.5 for *Fraxinus*, respectively, for both treatments. None of the groups were significantly different for *Fraxinus* (*P* > 0.5); similarly, all groups for *Celtis* were not significantly different, but the “low DF low RR,” which showed a significant reduction in root growth.

The correlations between DBH and total larger root diameter in 2017 were strong for both species (*r* ^2^ = 0.7 for *Celtis* and *r* ^2^ = 0.5 for *Fraxinus* for both treatments). Damage treatments did not have a significant (*P* > 0.5) effect on *Fraxinus* correlations, while the damage treatment combination of low DF/high RR significantly and positively affected *Celtis* abundance of roots (**Figure 8**).

## Discussion

### Multiple Damage Effects Are Not Simply Cumulative

Multiple damages did not have a simple cumulative effect on growth contrary to our first hypothesis. In most cases, they led to a less negative effect on tree growth than expected. In fact, the maximum damage treatment (DF 75%, RR 75%, and SD) did not have the most negative effect on stem and root growth for either species. Above- and below-ground woody production was decreased by all damage treatments for *Fraxinus* trees, but this decrease was relatively independent of treatment intensity and similar in all 18 damage treatments. In contrast, *Celtis*, which was experiencing a growth decline when damage treatments were applied, showed only a slight both positive and negative growth responses to treatments.

Interestingly, the effect of increasing intensity or number of damage treatments applied did not show a linear cumulative pattern. The effects of low or high intensity damage to only roots or the foliage did not affect the overall tree growth differently compared to the combined root and foliage damage treatments. Similar results can be found in urban oak where compensatory pruning was administrated following root loss to reduce water demand and crown dieback (Watson, 1998). Paired treatments did not decrease growth further; rather, it was similar to the reduction caused by a single treatment of defoliation or root reduction (Watson, 1998). This may indicate a synergistic effect of the damage treatments, inducing physiological responses that protect the trees from the second type of damage (Rennenberg et al., 2006; Bansal et al., 2013). It may also indicate that, in response to any significant stress, trees rapidly stop their growth to reallocate resources (i.e., mainly C from their photosynthesis or reserve pools) to cope with maintenance respiration, tissue reconstruction, or new tissue production (Körner, 2003; Muller et al., 2011; Körner, 2015).

### *Celtis* and *Fraxinus* Did Not Respond Similarly to the Damage Treatments

The different types, intensities, and combinations of damage treatments affected *Celtis* less than *Fraxinus*, 2 years after the last application, contrary to our second hypothesis. Moreover, trees treated with stem damage showed a greater stem growth compared to the control (**Figures 4** and **7**). This increased growth could be explained by a reaction to the injury and consequent compensatory growth to maintain tree viability, especially in trees already showing a decline in growth. Also, following stem damage like girdling, carbohydrates tend to accumulate in the upper parts of the tree (Winkler and Oberhuber, 2017), allowing above-ground compartments to be insensitive to or even to benefit from such treatment. Similarly, the low defoliation treatment in combination with stem damage had a boosting effect on both stem and root growth (**Figures 4** and **7**). In fact, the inclusion of stem damage with other damage treatments tended to reduce the negative effects of these other treatments, presumably due to the triggering of compensatory growth as has been shown in earlier studies (Mcnaughton, 1983; Gill, 1992; Watson, 1998). However, the small growth response of *Celtis* to any combination of treatments should take into account the low growth trend shown by this species following replanting (**Figure 1A**), and the additional stresses did not worsen the already observed decline. This would reinforce the hypothesis that under stress or with tissue damage, this species expresses a particularly high control of resource allocation to its C sinks to maintain its carbon balance (Hoch, 2015). Indeed, under stress, *Celtis* seems to be able to minimize growth and avoid mortality, sustaining “minimum growth levels” for several years. Such strategy may allow for the preservation of scarce resources and provide the capability of dealing with stress and associated secondary metabolism (Muller et al., 2011; Hartmann and Trumbore, 2016).

*Fraxinus*, which recovered well from replanting (**Figure 1B**), reacted more negatively to all treatments but for a shorter period of time compared to *Celtis* (**Figures 1** and **4**). The trees reacting to treatments, which combined similar levels of defoliation and root reduction, did not fare better than those following a single damage treatment. Growth showed a similar reduction when affected by single damage treatments compared to multiple damage treatments. Only trees undergoing stem damage, or stem damage paired with low defoliation, were not significantly different from the control. Roots showed a significant growth reduction with all damage treatments regardless of the type, intensity, or combination of damage, except in the trees that experienced only stem damage. This result refutes the hypothesis that damage to both above- and below-ground parts can minimize the negative impact on growth by balancing relative activity occurring both above- and below-ground. One should rather acknowledge that trees need to deal with each stress independently perhaps even simultaneously (see next section below). Hence, there is no benefit, nor apparent disadvantage, in trying to compensate for above- or below-ground damage by a similar reduction above- or below-ground.

### Damage Treatments Are Time Dependent

In answer to our third question, the three main damage treatments influence on growth shifted in importance and intensity in the years following their application. For stem growth, defoliation had a strong effect right after the damage treatment, while root reduction showed the highest negative effect in the long term (**Figures 5** and **6**). Stem damage was the factor with the lowest influence on stem growth, while it had a relatively strong effect on root growth. On the contrary, all damage treatments had equal weight in the contribution to root growth, and their effects remained constant in the years following the treatments. *Fraxinus* roots were the most affected by stem damage in the first year after treatment and equally affected by all three treatments thereafter. *Celtis* roots were affected most by defoliation in the first 2 years after damage treatment, while root reduction drove the differences in growth afterward.

In some cases, defoliation may reduce tree growth, or, on the contrary, photosynthetic up-regulation reactions may compensate for the loss of foliage resulting in a smaller impact on growth (Pinkard and Beadle, 1998; Vanderklein and Reich, 1999; Eyles et al., 2009; Li et al., 2016). In our study, *Celtis* stem growth rate had little response to defoliation, since growth of this species was actually already affected by other unknown factors. Nevertheless, there are numerous examples showing the lack of effect of low intensity defoliation on tree growth. For example, trees can compensate for a 25% or more defoliation treatment, showing no changes in concentration of nonstructural carbohydrates (Körner, 2003; Boege, 2005; Würth et al., 2005). A crown removal of 50% in Eucalyptus species showed no significant reduction in height and diameter increments over a 2-year period (Alcorn et al., 2008; Alcorn et al., 2013), indicating the great capability of trees to maintain above-ground growth rate from their reserve pools despite loss of foliage. On the contrary, *Fraxinus* stem growth responded to defoliation, by showing reduced growth rate initially, but recovering in the second year after the treatment, and remaining consistent in the following years (**Figure S1**). This compensation response after defoliation has already been reported and associated with the enhancement of photosynthetic efficiency after leaf loss and movement of resources to storage (Pinkard and Beadle, 1998; Quentin et al., 2012; Liu et al., 2019). The null and low root reduction and defoliation treatments showed a similar contribution to *Fraxinus* stem growth, while 75% of treatments showed the lowest contribution in the first 2 years after the treatment (**Figure 6**, **Figure S1**). Interestingly, in 2014 and 2015, high defoliation had a higher contribution to growth than the treatments of lower intensities.

In all cases, null and low root removal treatments showed similar growth contribution. Therefore, to maintain growth, low root removal might have a positive effect on remaining root activities, due to compensatory mechanisms enhancing water and nitrogen utilization efficiency (Blake, 1983; Ferree et al., 1999). However, this enhancement seemed to be insufficient or absent under higher root removal. Similarly, studies of root removal treatments on *Cunninghamia lanceolata* have also indicated multiple compensatory responses associated with root pruning, although above-ground biomass production was significantly negatively associated with increasing root removal (Dong et al., 2016). Trees treated with root removal exhibited higher water use efficiency, both right after the treatment and in the long term, along with an increase in fine root production. Root pruning at the 25% level seemed to be the most effective treatment to enhance photosynthetic nitrogen-use efficiency and stem dry mass accumulation (Dong et al., 2016).

### Damage Treatments Do Not Affect Above- and Below-Ground Growth Relationships in the Long Term

Surprisingly, the negative effects of the damage treatments were less pronounced than expected, showing no dramatic reduction when compared to the control, both above- and below-ground. Fine root production found near the stump increased in some of the damaged *Celtis* trees, especially those with the low root damage treatment. However, *Fraxinus* did not show any significant increase in fine root production in relation to the various damage treatments. The correlations between DBH and root production also show only minor differences among the damage treatments, and only the low DF/high RR showed a significant reduction for *Celtis*, probably due to a shift in resource allocation to a higher production of small roots and structural roots. The lack of significant differences between DBH and the larger root area produced in 2017 suggests that although these two species have different strategies when reacting to damage treatments and different growth rates and vitality, they both show that the impacts of damage treatments are not cumulative on tree growth.

Nonetheless, these results should be considered in light of the very different growth patterns of *Celtis* and *Fraxinus* in the growing conditions of this experiment, regardless of the treatments applied. Where *Celtis* showed low annual increments, *Fraxinus* showed high annual increments and a larger root system (Gucker, 2005; Gucker, 2011). Whereas *Celtis* control trees had reached an average of 8 cm DBH in 2017, *Fraxinus* reached a DBH of 11 cm, showing a 37.5% larger stem (**Figure 7**). Greater differences were found at the root level where *Fraxinus* grew 167% more roots than *Celtis*, although *Celtis* had a higher production of fine roots (on average 5% vs. 30%–40% of area covered by fine roots). In the event of a damage treatment, *Fraxinus* tended to keep the entire crown alive while sacrificing stem growth for a short period of time, whereas *Celtis* tended to sacrifice crown development and overall growth in order to maintain minimal stem and root increment (**Figure 3**). It is also interesting to notice how the correlation between roots produced and diameter growth did not drastically differ between the species and the treated groups (**Figure 8**), suggesting the capacity of both species to reach an equilibrium in above- and below-ground production, only 3 to 4 years after application of damage treatments. The only group showing a significantly higher root production was *Celtis* low DF/low RR. As discussed above, this is also evidence of a root-oriented compensatory mechanisms in *Celtis*, which stimulates root growth when low root removal is applied (Fare, 2014; Dong et al., 2016).

Furthermore, these species-specific differences need to be accounted for to understand any reaction to stress treatments. Overall, *Celtis* showed a system that focuses on the survival of the individual, possibly through a laborious and high-C demanding compartmentation and repair. One consequence is a long-term reduction or cessation of growth, to reduce structural growth sink and save C to cope with this stress response. The considerably low growth rates, probably associated with the shock of replanting, are consistent with trees that are capable of naturally growing in ravines and very rocky, unfavorable soils (Gucker, 2011). *Celtis* has been rated as the most damage-resistant species following hurricane damage (Xi and Peet, 2008), and a successful species in the rehabilitation of mining sites (Ashby et al., 1984). On the contrary, *Fraxinus* shows a high capacity for resource acquisition, compartmentalization after wounds, and growth recovery. This possibly explains its wide distribution across North America, ranging from southeastern Alberta, through central Montana to southeastern Texas, Florida, and up the east coast to Halifax (Kindscher and Holah, 1998). Although this species shows good climate adaptability, it is most often described in association with riparian areas, floodplains, and swamps, but is also found in areas that experience drought.

Finally, increasing stress does not imply a greater reduction in woody mass production. Although differences in their tolerance to damage were evident, neither species showed mortality with increasing stress. This indicates their suitability as urban trees. However, although this experiment shows the high capacity for adaptability of both *Celtis* and *Fraxinus*, it should be noted that physical damage still increases the occurrence of fungal and pathogenic infections, and vulnerability to insects, and therefore, injury should be avoided whenever possible (Clair-Maczulajtys et al., 1999). These conclusions do not take into account the emerald ash borer, which is posing a serious threat to *Fraxinus* health and vitality, and call into question the recommendation of planting *Fraxinus* in the urban setting (Klooster et al., 2018).

## Conclusion and Study Limitations

Both in vigorous or declining trees, multiple damage treatments do not have a cumulative effect on growth. Furthermore, for the vigorous *Fraxinus*, low intensity damage treatments stimulated growth after one or two growing seasons, which allowed a compensatory growth and at times an enhanced growth compared to control trees. This highlights the fact that damage can have a negative effect only above a certain threshold. Moreover, when surveying tree health, monitoring of damage impact should thus be done not only in the short term, but also years after damage occurs.

This experiment attempted to disentangle the effect of three types of damage commonly occurring in urban environments, at different levels of intensity both alone and in combination. However, there are three main limitations to the extrapolation of these results to urban trees. First, our sample trees were young, and it is difficult to extrapolate our results to a more advanced growth stage when growth and reserve storage change considerably. However, although direct comparisons between young and old trees’ reactions to damage were not carried out, it has been seen that growth of mature trees is significantly negatively affected by defoliation, which, for example, affected 80-year-old aspen for 2 to 3 years after the loss of foliage (Perrette et al., 2014), or major root removal (Dujesiefken and Stobbe, 2002). Mature spruce and beech trees showed a 3- or 4-year time lag before recovering from root trenching (Pretzsch et al., 2016). Second, 3 years of observations is a short period for evaluating the full effects of damage on trees. However, long-term experiments of this type are rare or nonexistent, so this study is a first step toward gaining a better understanding of the complexity of these damage treatments on tree growth. Third, our trees were growing in a rich and open agricultural field; it is not representative of a typical urban growing environment: the trees had plenty of water and space to grow and did not suffer from any pollution or high temperatures (Birkmann et al., 2010; Moser-Reischl et al., 2018). Although these growing conditions did not include common stresses that species can encounter in highly urbanized conditions, the differences in the growth trend of the control trees did highlight fundamental differences in vigor, showing a slow-growing *Celtis* and a fast-growing *Fraxinus*. Therefore, *Celtis* results reflect a “nonvigorous” population of trees, which may have been different if this species had not been experiencing a growth decline. Although no direct factor could be identified to justify *Celtis* low growth rate, it might be related to the shock of replanting in 2009, and roots system show a less clear pattern in reduction due to their larger variability.

This study did not intend to be an exhaustive compendium of species’ reactions to mechanical stress, but rather an unprecedented experiment aimed at assessing the effects of a large number of treatments and providing a unique insight into common reactions of urban tree species to mechanical stresses. Further experiments are needed to evaluate the threshold of stress treatments that shift the balance between positive reaction, a sustained negative impact, and growth collapse. This threshold is species-specific. In this study, *Fraxinus* growth was equally affected by all treatments regardless of intensity, whereas *Celtis*, while already declining at the time of planting, showed no enhanced negative impact on growth after the application of treatments and even marginally benefited from some specific low levels of damage. However, in this experiment, we could not provide further clarification about tree mortality; therefore, future research and long-term monitoring in urban environments are needed. Finally, both species, especially at a young stage, although through different strategies, seem to be particularly resistant and resilient to various levels and combinations of above- and below-ground damages often found in urban settings. Such high resistance and resilience to single or multiple damages do not seem to occur at the detriment of decreasing levels of reserve found in the trees (Ramirez et al., 2018). Further studies done on more tree species under more stressful conditions such as found in urban areas are necessary to generalize our results.

## Data Availability

The datasets for this manuscript are not publicly available because data still in use for further publications. Requests to access the datasets should be directed to valentina.vitali89@gmail.com. 

## Author Contributions

VV produced and processed the roots data, performed the analyses on the roots and stem data, and wrote the paper; JR and GP supervised and carried out the experimental procedures and helped with the evaluation and discussion of the results; CM and JR conceived the study idea, developed the study design, and cowrote the paper; and AP and SD also cowrote the paper.

## Funding

This research was supported by the NSERC/Hydro-Quebec research chair on tree growth control and by a scholarship from the Quebec research funds for nature and technology.

## Conflict of Interest Statement

The authors declare that the research was conducted in the absence of any commercial or financial relationships that could be construed as a potential conflict of interest.
