# Supplementary material for: Complex Above- and Below-Ground Growth Responses of Two Urban Tree Species Following Root, Stem, and Foliage Damage—An Experimental Approach
Source: Front Plant Sci. 2019 Sep 18;10:1100. doi: 10.3389/fpls.2019.01100 (PMC6759508; doi:10.3389/fpls.2019.01100)
Supplement: Supplementary file 1 [file DataSheet_1.docx]

**Supplementary material**.

**Table S.1** Fitted mixed models showing the effect of treatments on tree ring growth of *Celtis* and *Fraxinus* stems and roots during the years 2012-2015. The treatments are described as SD=stem damage50% stem removal), RR=root removal and Df= defoliation indicated as Low=37% damage and High= 75% damage. Mixed models are based on 436 for *Celtis* and 324 observations for *Fraxinus* (r² ~0.1 for all models). The significant effect (*p, 0.1, **p<0.05; ***p,0.01Bold values) of each treatment references to the distance from the growth shown by the control group.

| Treatment | Celtis  Stems |  | Roots |  | Fraxinus  Stems |  | Roots |  |  |
| --- | --- | --- | --- | --- | --- | --- | --- | --- | --- |
| Low DF | 0.028 |  | -0.69 |  | -2.45 | *** | -3.313 | *** |  |
| Low RR | 0.146 |  | -0.01 |  | -1.655 | * | -2.492 | *** |  |
| High DF | -0.383 |  | -0.62 |  | -1.893 | ** | -1.547 | ** |  |
| High RR | -0.732 | * | -0.84 |  | -2.732 | *** | -3.326 | *** |  |
| SD | 0.793 | * | 1.274 | ** | -1.137 |  | -1.264 |  |  |
| Low DF SD | 1.883 | *** | -0.20 |  | -0.817 |  | -1.795 | ** |  |
| Low RR SD | 0.227 |  | -0.76 |  | -2.156 | ** | -2.375 | *** |  |
| Low DF Low RR | 0.975 | ** | 0.366 |  | -1.344 | *** | -3.142 | *** |  |
| Low DF High RR | -0.183 |  | -0.79 |  | -4.423 | *** | -3.869 | *** |  |
| LowDF Low RR SD | 0.674 |  | 2.284 | *** | -1.632 | * | -2.521 | *** |  |
| Low DF High RR SD | -0.0004 |  | -0.43 |  | -3.661 | *** | -2.155 | *** |  |
| High DF Low RR | -0.649 |  | -1.59 | *** | -1.365 | *** | -2.888 | *** |  |
| High DF SD | -0.311 |  | 0.640 |  | -2.334 | *** | -3.364 | *** |  |
| High RR SD | -0.556 |  | -0.88 |  | -2.431 | *** | -2.268 | *** |  |
| High DF Low RR SD | -0.31 |  | -1.23 | ** | -1.955 | ** | -2.491 | *** |  |
| High DF High RR | -1.109 | *** | -1.11 | * | -3.369 | *** | -2.787 | *** |  |
| MaxDamage | -0.695 | * | -1.42 | *** | -3.055 | *** | -2.225 | *** |  |
| Constant | 2.462 | *** | 2.832 | *** | 7.34 | *** | 5.092 | *** |  |


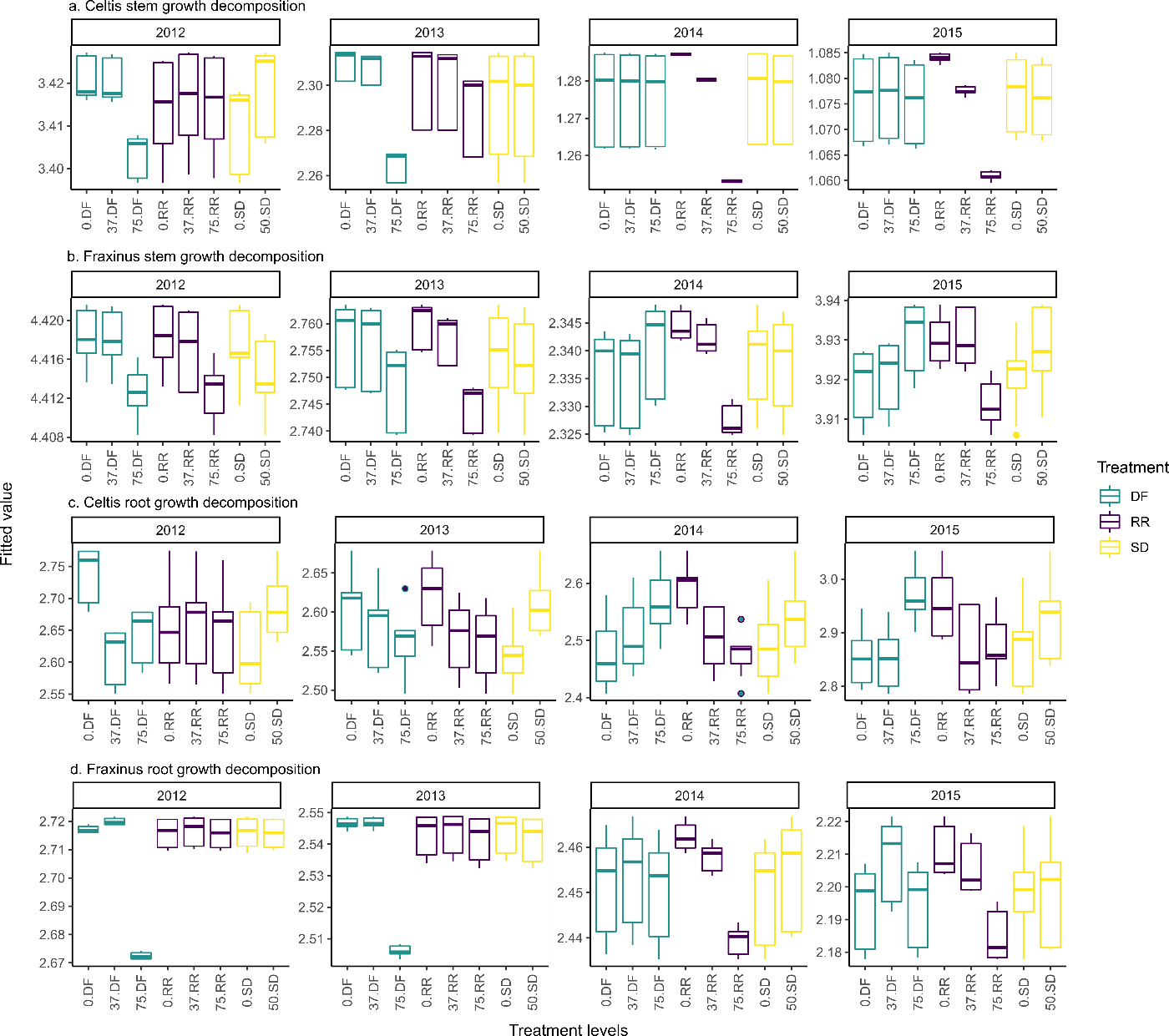


**Figure S. 1** Partial dependence plots showing tree ring growth decomposition between the different levels of treatments obtained by the GBM model for all the years after the treatments. Where DF= defoliation, RR = roots removal and SD = stem damage, 0=null treatment, 37=low intensity treatment, 75= high intensity treatment. Panel a. and b. for the stem growth decomposition of *Celtis* and *Fraxinus*, c. and d. for the root’s growth decomposition of *Celtis* *Fraxinus*. Scales are different in each panel to maximise the visibility of the differences between the treatment levels.
